# Network-based analysis of transcriptional profiles from chemical perturbations experiments

**DOI:** 10.1186/s12859-017-1536-9

**Published:** 2017-03-23

**Authors:** Francesca Mulas, Amy Li, David H. Sherr, Stefano Monti

**Affiliations:** 1 Division of Computational Biomedicine, Boston University School of Medicine, 72 E Concord St, Boston, 02118 USA; 2 Department of Environmental Health, Boston University School of Public Health, 72 E Concord St, Boston, 02118 USA

**Keywords:** Correlation networks, Toxicogenomics, Compounds similarity, Gene expression, Comparative analysis, Chemical perturbations

## Abstract

**Background:**

Methods for inference and comparison of biological networks are emerging as powerful tools for the identification of groups of tightly connected genes whose activity may be altered during disease progression or due to chemical perturbations. Connectivity-based comparisons help identify aggregate changes that would be difficult to detect with differential analysis methods comparing individual genes.

**Methods:**

In this study, we describe a pipeline for network comparison and its application to the analysis of gene expression datasets from chemical perturbation experiments, with the goal of elucidating the modes of actions of the profiled perturbations. We apply our pipeline to the analysis of the DrugMatrix and the TG-GATEs, two of the largest toxicogenomics resources available, containing gene expression measurements for model organisms exposed to hundreds of chemical compounds with varying carcinogenicity and genotoxicity.

**Results:**

Starting from chemical-specific transcriptional networks inferred from these data, we show that the proposed comparative analysis of their associated networks identifies groups of chemicals with similar functions and similar carcinogenicity/genotoxicity profiles. We also show that the in-silico annotation by pathway enrichment analysis of the gene modules with a significant gain or loss of connectivity for specific groups of compounds can reveal molecular pathways significantly associated with the chemical perturbations and their likely modes of action.

**Conclusions:**

The proposed pipeline for transcriptional network inference and comparison is highly reproducible and allows grouping chemicals with similar functions and carcinogenicity/genotoxicity profiles. In the context of drug discovery or drug repositioning, the methods presented here could help assign new functions to novel or existing drugs, based on the similarity of their associated network with those built for other known compounds. Additionally, the method has broad applicability beyond the uses here described and could be used as an alternative or as a complement to standard approaches of differential gene expression analysis.

**Electronic supplementary material:**

The online version of this article (doi:10.1186/s12859-017-1536-9) contains supplementary material, which is available to authorized users.

## Background

Network-based approaches to the analysis of social and biological networks have provided scientists with powerful tools for the intuitive visualization of complex processes and for the principled integration of multiple data sources. In genomics, network-based models – with genes represented by nodes and gene-gene interactions represented by edges – are inferred from experimental data or retrieved from manually curated repositories available online. Data-driven transcriptional networks, where a link between two nodes denotes a strong association between the corresponding gene expression profiles, are particularly useful as they represent a snapshot of the gene co-regulation in the experiment under study [1].

Within this context, scale free networks (SFN), in which the degree of connection of the member nodes follows a power law, have been widely used in the study of protein interactions [2]. The construction of these networks usually relies on the computation of gene-gene correlations across replicate experiments, and on the subsequent thresholding of the absolute correlation values so as to define as connected only those genes with correlation above a chosen threshold [3, 4]. Although this approach has proven extremely useful in identifying key hub genes in multiple biological conditions [5], the high sensitivity of the obtained networks to the choice of threshold raises questions about the reproducibility of the obtained results, as well as about their biological meaning [1, 4].

An alternative approach is to use Correlation Networks (CN), where all pairwise gene associations are considered, to avoid loss of information in those cases where the analysis focuses on the identification of groups of tightly connected genes (modules), rather than on the identification of single key nodes (hubs). While both types of graphs are referred to as *networks* in the following manuscript, it should be noted that the CN approach yields a fully interconnected (albeit weighted) graph, on which some of the topological indices developed for “standard” networks with sparse links are not applicable.

Regardless of the methodology used to infer the graph, network-derived gene modules can be investigated experimentally in order to gain insights into their biological function, or with the help of gene and pathway annotation resources. Additionally, the comparison of correlation networks from different conditions (e.g., different disease stages, or perturbations with different chemicals) may help identify modules whose connectivity is significantly altered in the compared conditions [6]. Connectivity-based comparisons may thus help identify “aggregate changes” that could be missed by standard methods of differential analysis comparing individual genes [7].

In this study, we describe the development of a network-based analysis pipeline and its application to gene expression datasets from chemical perturbation experiments, with the goal of elucidating the modes of actions of the profiled perturbations. We apply our pipeline to the analysis of the DrugMatrix dataset from the National Toxicology Program (NTP) [8] and the TG-GATEs dataset from the Japanese National Institute of Biomedical Innovation [9], two of the largest toxicogenomics datasets available, which contain organ-specific gene expression measurements for model organisms exposed to hundreds of chemical compounds with varying carcinogenicity and genotoxicity.

Evidence accumulated to date has shown that machine learning techniques can successfully be applied to infer chemical carcinogenicity (or genotoxicity) from expression profiles of in vitro and in vivo assays [10]. In our own previous work, we have shown that it is possible to infer highly accurate predictive models of chemical-associated long-term cancer risk from rat-based short-term toxicogenomics data, and to identify genes significantly associated with carcinogenesis [11]. Here, we aim to go beyond the inference of predictive models and the identification of single biomarker genes, towards the identification of gene modules or pathways significantly associated with the profiled chemical perturbations and the induced adverse phenotypes. We do so by comparing the connectivity of gene modules in the networks derived from the control samples (“Control network”) to those obtained from samples collected after the exposure to specific chemical compounds.

To this end, we reconstruct chemical-specific transcriptional networks, and show that by grouping chemicals based on the similarity of their associated networks we can identify groups of chemicals or drugs with similar functions and similar carcinogenicity/genotoxicity profiles. We also show that the in-silico annotation by pathway enrichment analysis of the gene modules with a differential connectivity (i.e. showing a gain or loss of connectivity for specific groups of compounds) can point to the main molecular pathways induced by specific chemicals.

While network-based differential analysis of gene expression profiles is not new, the novelty of our approach lies in the application of the approach to aggregate chemical perturbations by their network similarity, in our application of a bootstrapping-based statistical significance test, and in the module-based meta-analysis of the differential connectivity results across multiple perturbations. While in this study we focused on chemical-induced carcinogenesis and genotoxicity as the adverse phenotypes of interest, the approach we propose has broader applicability.

## Results

### Differential connectivity analysis of chemical perturbations

**Fig. 1:**
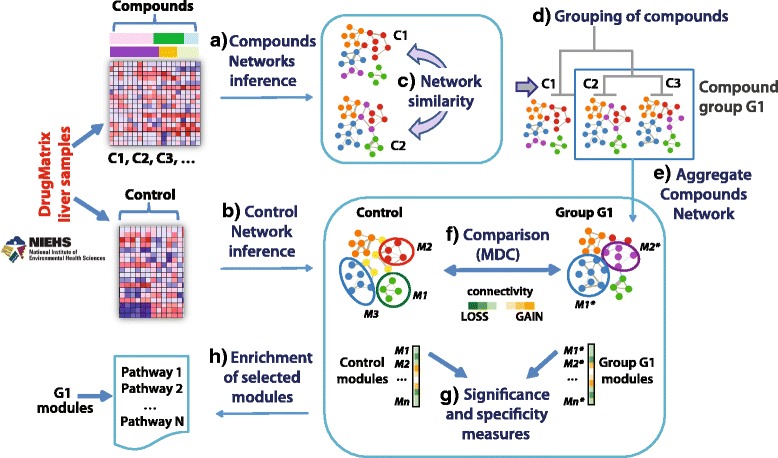
Analysis workflow. DrugMatrix liver samples are used to infer chemical-specific Compound Networks (a) and a Control Network (b). Similarity in terms of network structure is evaluated to identify groups of compounds, whose samples are pooled to infer Aggregate Compound Networks (c-e). Modules of tightly connected genes both in the Control network and in Compound Aggregate networks are identified and compared across conditions in terms of Module Differential Connectivity (MDC) (f). Modules with a change in connectivity that is highly specific to each compound group are investigated through pathway enrichment analysis (g-h)

As shown in Fig. 1, the network-based pipeline for differential connectivity analysis starts by inferring chemical-specific Compound Networks, obtained from samples collected after the exposure to specific chemical compounds (Fig. 1.a), and a network from the control samples, hereafter named “Control Network” (Fig. 1.b). Groups of compounds are then identified based on the similarity of their individual network structures. For each group, a new “Aggregate Compound Network” is inferred by pooling all the samples across the clustered compounds (Fig. 1.c-e). Next, modules of tightly connected genes are identified in each of the constructed networks and compared between conditions (i.e., control vs. compound group) in terms of Module Differential Connectivity (MDC) (Fig. 1.f). Given a module identified in one of the two networks under comparison (e.g., the aggregate compound network), the MDC score is computed as the ratio of the average connectivity across all the genes within the module in the aggregate network (numerator) and in the control network (denominator). This score represents changes of connectivity in the Compound group with respect to the Control (see Methods). MDC scores are computed for all modules in the networks, and tests of statistical significance and module *specificity* are performed to identify modules that will be further investigated through enrichment analysis based on pathway repositories and additional annotation sources (Fig. 1.g-h).

### Network comparison is sensitive to cross-dataset batch effects

Before applying the described pipeline, we sought to rigorously assess the reproducibility of the methodologies used. To this end, we carried out a systematic validation by comparing networks inferred from independent sets of samples profiling the same experimental conditions. This approach was used under the assumption that networks derived from the same experimental conditions should in theory yield very similar structures, with differences in connectivity only due to sampling variability. However, in practice many sources of “unwanted variation” can contribute to the measured differences, first among them differences due to non-reproducible experimental settings (batch effects). For this reason, we performed two types of validation experiments, aimed at identifying network inference and comparison methods showing a high reproducibility and robustness to possible batch effects.

First, networks derived from the control (i.e., unexposed) liver samples of two independently generated datasets (DrugMatrix and TG-GATEs) were compared in terms of the composition of gene modules and their observed changes in connectivity. For each of the modules identified from the TG-GATEs network, a correspondent module (i.e., a module composed of a highly overlapping set of genes) was found in the DrugMatrix network. The high significance of the overlap for all the modules (Fisher test, *p* < 10^-16^) confirmed that the two networks yielded the same structure and modules composition. However, a systematic deviation of the distribution of differential connectivity values from one was detected in multiple modules. A careful analysis showed that MDC values different from 1 were mainly caused by a difference in the distribution of correlation values between the DrugMatrix and the TG-GATEs datasets (Additional file 1: Figure S1.a). Furthermore, cross-dataset scaling or normalization could not eliminate these differences. Our evaluation points to the need for carefully assessing the methodologies and samples used for network inference and to adopt rigorous permutation-based statistical testing, in order to avoid interpreting artifacts or batch effects as actual differences in modules’ connectivity.

Secondly, the inference and comparison methods were evaluated on networks derived from independent sample subsets extracted from the same dataset (by a resampling approach, see Methods). In this case, we attained more reproducible results, with the distribution of MDC values correctly centered at 1, and with a lower variance when using the correlation network (CN) approach rather than the Scale-Free Network (SFN) approach (Additional file 1: Figure S1.b). An additional advantage of the simpler CN approach is that it does not require the selection of a threshold for the correlation values, a choice that is highly sensitive to the samples analyzed and strongly influences the subsequent calculation of MDC values.

Based on these results, the subsequent analyses were all based on a single dataset (the DrugMatrix, which includes a higher number of compounds), and the CN approach was selected as the network inference method of choice.

### Groups of similar chemicals can be inferred by network analysis

Using the CN approach, we analyzed how different groups of compounds affect the connectivity pattern of specific gene modules. First, we inferred the network from the non-treated liver samples, hereafter named “Control Network”. The Control Network was clustered into 60 gene modules, with sizes ranging from 21 to 551 genes.

**Fig. 2:**
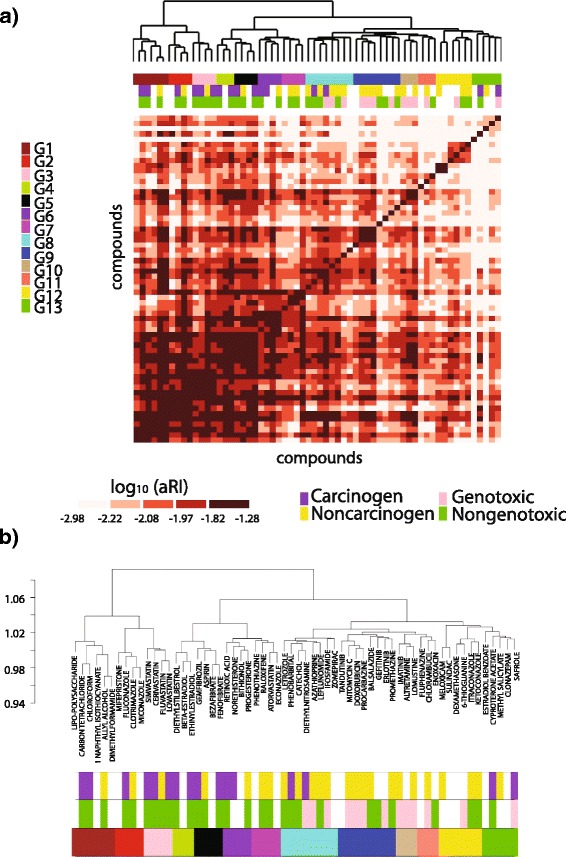
Compounds aggregation. Similarity of 62 chemical compounds based on adjusted Rand Index (aRI). **a.** Heatmap of aRI and grouping of compounds with similar networks structure. **b.** Zoom-in on the compounds grouping

Next, chemical-specific Compound Networks were inferred for each of the 62 chemicals for which at least ten replicate experiments (animals) were available. Although a comparison of modules connectivity in Control and Individual chemicals-related networks was carried out (see Methods section and Additional file 1: Figure S3 a-b), we aimed at identifying chemicals with similar network structure, which were then grouped to infer “Aggregate Compound Networks”. This step had two main goals: i) to study how well groups of chemicals with similar known features (e.g., similar mechanism of action) can be identified through networks; and ii) to increase the sample size available for network inference. For the aggregation of the chemicals, the Compound Networks were compared pairwise based on the similarity of their respective modules as measured by the adjusted Rand index (aRI) [12]. The aRI is a score specifically devised for the comparison of clustering results even when the two networks have different number of clusters (i.e., modules). Hierarchical clustering was then applied to the matrix of aRI’s to induce a similarity-based partial ordering and grouping of the chemicals (Fig. 2.a).

The aRI-based clustering yielded a clear separation between non-genotoxic carcinogens (left sub-dendrogram in Fig. 2.b) and genotoxic non-carcinogens (right sub-dendrogram). Of notice, genotoxic compounds were not as well separated when we applied alternative, more standard clustering approaches, such as one based on the direct similarity of the chemicals’ expression profiles, and one based on the chemicals’ shared interacting proteins as annotated in public databases (Additional file 1: Figure S2.a-b).

Dynamic tree cutting [13] was applied to the aRI-based dendrogram with the parameters chosen so as to yield clusters with adequate sample size and sufficiently homogeneous sets of perturbations. By this procedure, 13 chemical groups were identified and analyzed to assess their internal similarity as determined by multiple criteria. According to DrugBank annotation, 11 of the 13 chemical groups showed a high overlap in the main pharmacological action of their member chemicals as annotated in the STITCH database [14] (Table 1). As an example, group G3 includes only hypolipidemic drugs classified as “Statins” present in the dataset, and it is separated from group G5, which is mainly composed of “Fibrates”, another class of antihyperlipidemic agents. Remarkably, some of these groups were not identified by standard gene expression-based analysis. These include groups G1 and G8, whose chemicals were separated into multiple clusters by Dynamic tree cutting of the dendrogram shown in Additional file 1: Figure S2.a, obtained by gene expression similarity. Compounds within these two groups were indeed significantly similar, as measured by the overlap of their interacting proteins (Table 2). Overall, a significant internal similarity was observed in 7 of the 12 groups for which protein annotations were available from public databases (*p* < 0.01). High overlap of shared side effects was also observed, although only two compound groups showed statistical significance by the permutation-based approach, due to a large number of side effects that are common to most of the compounds available in the SIDER database [15].

**Table 1:** Compound groups. Main functions retrieved through the STITCH database

Group ID - Main Function	Compounds	Functions
G1 - Solvents	carbon tetrach	cleaning agent
	chloroform	solvent
	dimethylformamide	solvent
	allyl alcohol	alcohol
	1-naphthyl isothiocyanate	preservative
	lipo-polysaccharide	endotoxin
G2 - Antifungals	clotrimazole	antifungal
	fluconazole	antifungal
	miconazole	antifungal
	mifepristone	steroid
G3 - Statins	cerivastatin	statin
	fluvastatin	statin
	lovastatin	statin
	simvastatin	statin
G4 - Estrogens	diethylstilbestrol	estrogen
	beta-estradiol	estrogen
	ethinylestradiol	estrogen
G5 - Fibrates	aspirin	anti-inflammatory
	fenofibrate	fibrate
	gemfibrozil	fibrate
	bezafibrate	fibrate
G6 - Steroids	bithionol	photosensitizer
	norethisterone	progestogens
	progesterone	progestogens
	retinoic acid	progestogens regulator
G7 - n.c	atorvastatin	statin
	econazole	antifungal
	raloxifene	estrogen
	phenothiazine	antipsychotic
G8 - Anti-Cancer	catechol	benzenediols
	azathioprine	cancer drug
	ifosfamide	cancer drug
	leflunomide	antirheumatic
	letrozole	cancer drug
	phenobarbital	anticonvulsant
	zomepirac	antipyretic
	diethylnitrosamine	tumorigenic
G9 - Chemotherapeutics	doxorubicin	chemotherapeutic
	procarbazine	chemotherapeutic
	promethazine	neuroleptic
	mitomycin C	chemotherapeutic
	gefitinib	cancer drug
	erlotinib	cancer drug
	tandutinib	antineoplastic
	balsalazide	anti-inflammatory
G10 - Alkylating, Cancer	altretamine.	alkylating
	lomustine	cancer drug
	imatinib	cancer drug
G11 - n.c.	chlorambucil	cancer drug
	enoxacin	antibacterial
	fluphenazine	antipsychotic
G12 - Anti-Inflamm/ Fungal	dexamethasone	anti-inflammatory
	itraconazole	anti-fungal
	ketoconazole	anti-fungal
	meloxicam	anti-inflammatory
	sulindac	anti-inflammatory
	6-thioguanine	anti-inflammatory/cancer
G13 - Antiseptics, Estrogens	clonazepam	anxiolytic
	cyproterone acetate	estrogenic
	estradiol benzoate	estrogenic
	safrole	antiseptic
	methyl salicylate	antiseptic

**Table 2:** Groups’ internal similarity by multiple criteria

Group	Function	Significance of interacting proteins	Significance of common side effects
G1	Solvents	6.61E-33 *	NA
G2	Antifungals	4.45E-19 *	8.47E-11
G3	Statins	1.69E-33 *	1.29E-145 *
G4	Estrogens	NA	NA
G5	Fibrates	1.02E-56 *	6.60E-20
G6	Steroids	1.56E-06 *	NA
G7	n.c. (estrogens, antifungals)	0.0002	1.77E-05
G8	Anti-Cancer	1.69E-06 *	7.91E-16
G9	Chemoterapeutics	9.79E-05	1.78E-32 *
G10	Alkylating, Cancer	0.0001	2.79E-07
G11	n.c (anti-cancer, estrogens)	0.0012	4.80E-15
G12	Anti-Inflamm/ Fungal	1.28E-18 *	2.91E-18
G13	Antiseptics, Estrogens	0.0004	NA

*NA* = no annotations were found in CTD or in SIDER. * = lower p-value cannot be obtained by chance (permutations)

In summary, our analysis showed that the comparison of network modules by aRI yields groups of chemicals with similar carcinogenicity-genotoxicity profiles in terms of pharmacological action, interacting proteins, and side effects.

### Module differential connectivity highlights chemicals’ modes of action

Samples related to the 13 groups identified were used to infer Aggregate Compound Networks, each representing the partial correlation among genes across all replicate experiments from the group of compounds considered. As shown in Fig. 1, we aimed at comparing the Control Network with multiple perturbations by analyzing the changes in gene modules connectivity. This analysis was repeated twice, first using the modules identified in the Control Network, and then using the modules identified in each of the Aggregate Compound Networks.

#### Control network-centered analysis

**Fig. 3:**
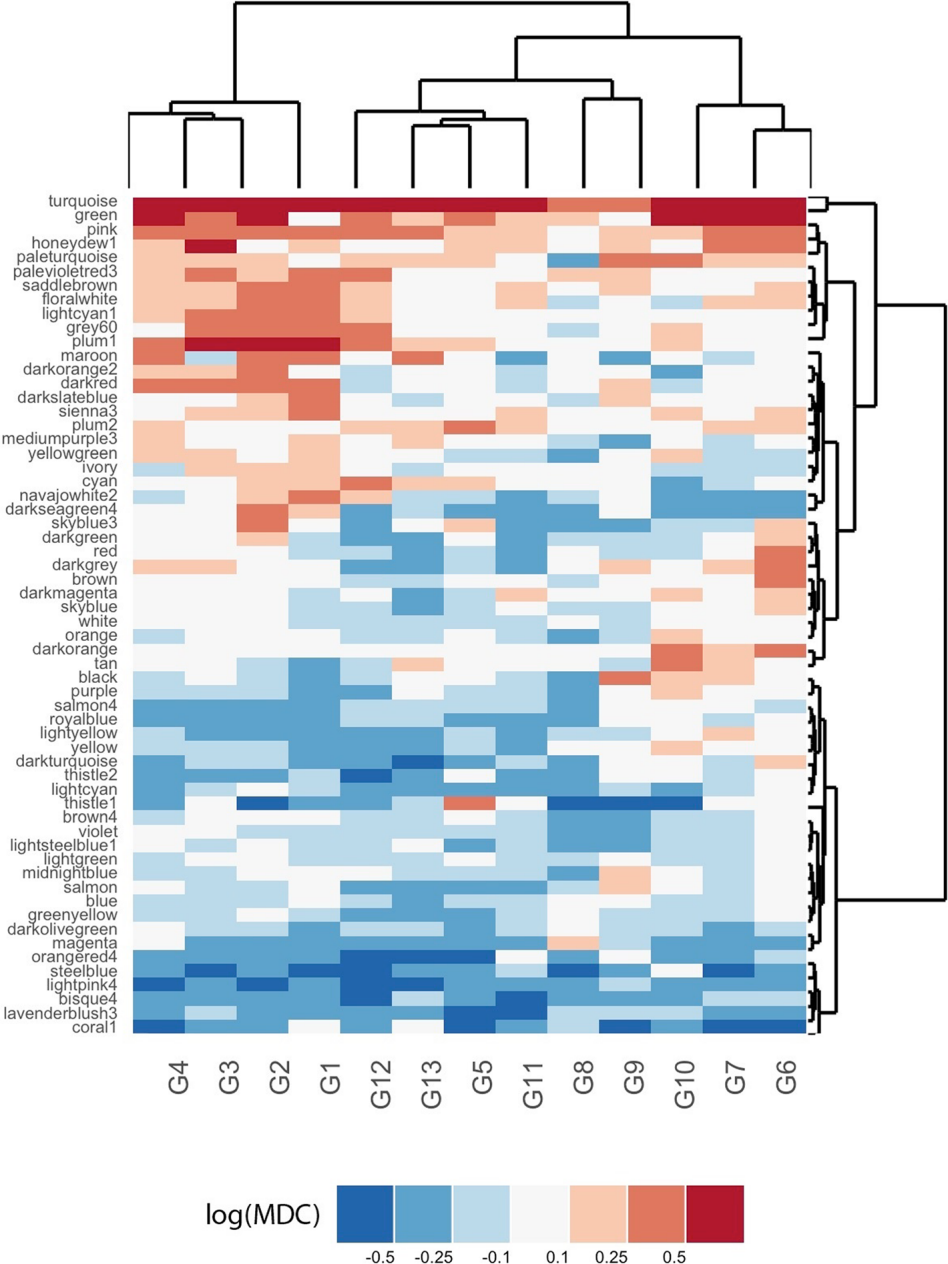
Gain and loss of connectivity of Control modules. Differential connectivity of 60 Control modules (rows) induced by 13 groups of compounds (columns). The heatmap is color-coded according to the MDC values, with blue and red indicating a loss and a gain of connectivity, respectively

For each of the modules identified in the Control Network, the MDC score was computed to measure the change in connectivity (gain or loss) among the module’s genes due to the action of each group of chemicals. Significance of the MDC values was assessed by a bootstrap approach, whereby a confidence interval for each MDC is estimated by performing network inference on bootstrapped (i.e., sampled with replacement) versions of the original dataset, as detailed in Methods and in Additional file 1: Figure S4. An alternative, permutation-based significance testing procedure was also evaluated, whereby p-values are obtained by computing MDC values for random sets of genes, with sizes equal to each module under study. However, this approach was not sufficiently stringent, yielding an excessively large number of likely false positive results (data not shown). The bootstrap approach identified a more parsimonious set of highly significant MDC values (Additional file 1: Figure S5). Figure 3 summarizes the Control Network-centered results. Since the modules are defined in the control network, hence are the same for each pairwise comparison, the results can be represented as a matrix and an associated color-coded heatmap, with each row corresponding to a Control Network module, and each column corresponding to a compound group. Several modules manifest a remarkable change of connectivity, as captured by their MDC scores, and as confirmed by their estimated q-values (Additional file 1: Figure S4).

Of notice, the “turquoise” module was the only group showing a highly significant gain of connectivity for all the groups of compounds analyzed. This result can be explained by the remarkably high number of genes included in this module (551), if compared to the median size of all the modules, equal to 93. Since chemical perturbations induced a global increase of the connectivity of the entire network (Additional file 2: Table S1), large size modules are more likely to show the same pattern, i.e. a gain in the connectivity when compared to the Control Network. Moreover, biological annotation of this module showed a variety of different pathways, suggesting that compounds with diverse function could have an effect on this group of genes.

**Fig. 4:**
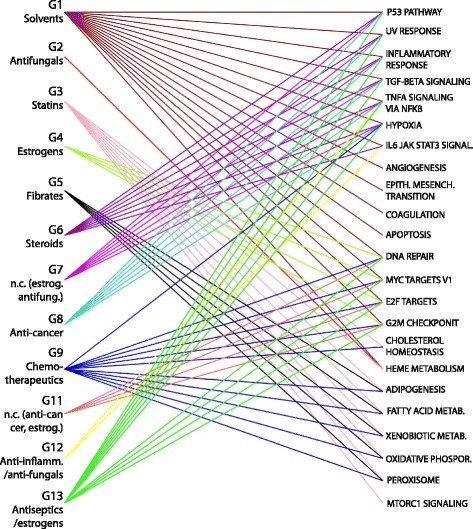
Enrichment of specific Control modules. Bipartite graph representing associations between compound groups and enriched Hallmarks gene set corresponding to specifically altered modules extracted from the Control Network

In order to focus on compound-specific effects, we computed a “Specificity Score” for each Control module [16]. The specificity score quantifies the uniqueness of a gain or loss of connectivity to a given compound group. Briefly, for a given group of compounds and a given module, the differences in MDC between that group of compounds and all the other groups are computed. Specificity is then defined as the sum of all the differences, with higher values identifying modules with a high MDC absolute value relative to all the others. Modules with scores exceeding a top percentile of the distribution of Specificity values were subject to enrichment analysis and significant pathways were selected with FDR-corrected p-values. Additional file 3: Table S2 shows enrichment analysis results of the best-ranked specific modules for each compound group, and a bipartite graph in Fig. 4 is used to graphically represent the obtained associations between compound groups and enriched gene sets. All groups except one (G10) showed at least one top specific module significantly enriched for Hallmarks gene sets (Enrichment FDR-corrected *p*-value < 0.25).

#### Aggregate network-centered analysis

Taking an approach complementary to the one adopted in the control network-centered analysis, here a set of gene modules is defined *for each* of the aggregate compound networks. That is, for each aggregate network, a set of densely connected modules is identified, and their change of connectivity with respect to the control network is calculated by MDC. Since a potentially distinct set of modules is identified in each aggregate network, this precludes the representation of the differential connectivity analysis results across the aggregate networks in matrix form.

**Fig. 5:**
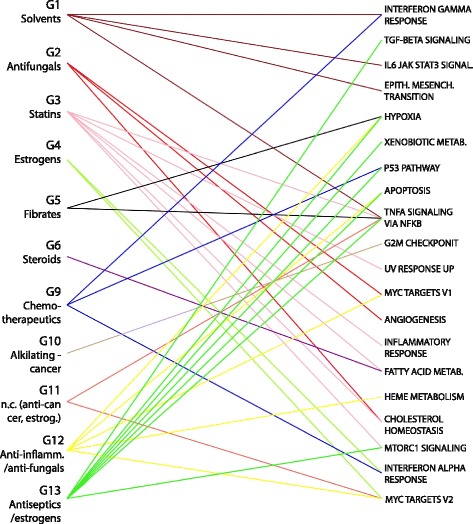
Enrichment of specific Compounds-related modules. Bipartite graph representing associations between compound groups and enriched Hallmark gene sets corresponding to specifically altered modules extracted from each Aggregate Compound Network

In this analysis, we first identified *high frequency* (HF) modules as those modules for which similar grouping of genes (i.e., similar composition) was found across multiple aggregate networks (Fisher test, *p* < 0.01). A Specificity Score was then computed to highlight those with MDC values specific to a particular compound group. We next identified, *low frequency* (LF) modules, i.e., modules whose composition was unique to only one or few Aggregate Networks. Both HF and LF modules are reported in Additional file 4: Table S3, and graphically represented in Fig. 5, where Hallmarks gene sets have been used to investigate their biological function. Taken together, the findings described below confirm that the approach is capable of identifying known modes of action [10], and of grouping compounds based on their coordinated effect on molecular pathways.

In addition, a comparison of our network-based analysis results with those from standard differential expression analysis highlighted the complementarity of the two approaches. Differentially expressed genes identified as belonging to the “Perturbational transcriptome” in our previous study [11], and their correspondent enriched Hallmarks, were evaluated in terms of their overlap with genes and gene sets identified by the present approach. First, each of the Control and Compounds-related modules shown in Additional file 3: Table S2 and Additional file 4: Table S3 was scored for its enrichment in terms of differentially expressed genes. As expected, a majority of the modules (26 out of 32) were significantly enriched for differentially expressed genes (Fisher test, *p* < 0.01). Additional file 5: Table S4 shows a summary of this analysis where all genes contained in at least one of the modules identified are compared with the Perturbational transcriptome. Despite the significant overlap yielded by the two approaches, a considerable number of genes were identified only by one of the methods, pointing to the complementarity of the approaches. Interestingly, many of the genes and Hallmarks identified only with the network-based approach were associated to pathways previously implicated in mediation to chemical responses, and described in the following section, including Heme Metabolism, Myc targets, and inflammation signaling.

## Discussion

The following sections aim to discuss the main biological findings resulting from both Control-centered and Compounds-centered analyses.

### Alcohol-induced liver inflammation

As shown in Fig. 4, G1 has a specific effect on a high number of pathways, mostly associated with inflammation and tumorigenesis. Given that liver samples have been considered in this study, the significant impact of this group on the entire pathway set could be explained by the high number of alcohols included in group G1. In fact, alcohol-mediated activation of inflammation signaling pathways in the liver is known to increase tumorigenesis in mice and to activate pro-inflammatory cytokine, such as tumor necrosis factor alpha (TNF-α), interleukin 6 (IL-6), and nuclear factor kappa B (NFκB) [17]. This liver damage response has been reported for several members of the “G1-Solvents” group, including allyl alcohol [18], Lipopolysaccaride, known as an endotoxin, [17], and chloroform [19]. In particular, chloroform has been associated to an anti-inflammatory action, possibly explaining the loss of connectivity of module “coral1” in Additional file 4: Table S3. Concordantly, TNF-α has been reported as showing a remarkable functional duality, being strongly engaged both in tissue regeneration/expansion and destruction [20].

### Hypolipidemic compounds induce cholesterol metabolism and inflammation

Groups G3 (Statins) and G5 (Fibrates), both including hypolipidemic compounds, show a specific alteration of modules related to cholesterol and fatty acid metabolism. In particular, the highest Specificity score in Additional file 3: Table S2 is obtained by the Fibrates on module “thistle1”, enriched for Fatty Acid Metabolism. The MDC values color-coded in Fig. 3 show that while all the other groups of chemicals cause a LOC of the “thistle1” module, groups G5 and G3 are the only ones to produce a GOC, with a higher MDC obtained by Fibrates. Statins have a more significant effect on the module “honeydew”, enriched for cholesterol homeostasis (Additional file 4: Table S3), which has not a high specificity ranking for the Fibrates, confirming different actions of those two distinct classes of drugs. Statins are also associated with stress response pathways, including oxidative phosphorylation, UV response and activation of TNF-α in both Control (Fig. 4) and compounds-related modules (Fig. 5). While hypoxia-inducible factors were found to play a role in the inhibition of cholesterol synthesis [21], an inflammatory response of the liver has not been clearly reported in the literature. However, the indication of liver damage as a rare side effect by FDA might suggest that tissue-specific network analysis could capture most of the possible mechanisms induced by drugs exposure.

### Effect of estrogens, steroids and cancer drugs on cellular replication

Both modules related to estrogens and to cancer treatment have an effect on the connectivity of module “maroon”, enriched for pathways related to cellular replication. While this module is gaining connectivity as an effect of proliferation-inducing drugs contained in group G4 (estrogens), a loss of connectivity is observed for G9 (chemotherapeutics) and G11, pointing to the disruption of the cellular replication machinery caused by anti-cancer drugs. The gain of connectivity of G2M checkpoint induced by G10 (Alkylating-cancer) and p53 pathway in the compound-related modules (Fig. 5) also confirms known mechanisms in the treatment of tumors and DNA damage [22].

The loss of connectivity of inflammation-related pathways observed on G6 perturbation could be explained by the known action of some steroids-related compounds. In particular, a negative correlation with retinol and liver fibrosis has been previously reported [23, 24].

Another interesting effect of one of the steroid compounds is revealed by enrichment analysis of modules altered by group G2, mainly composed of antifungals. Our results show an effect of this group on coagulation pathways, observed in the Control modules and supported by the literature [25], as well as an effect on regeneration-related pathways, observed in the Compounds-related modules. Remarkably, this group also contains mifepristone, a steroid compound that was also shown to have an effect on heme pathways. In addition, significantly lower effects on coagulation pathways were shown for other steroid compounds with pharmacological action similar to mifepristone [26]. This finding highlights a similar action of the compounds included in G2, despite being assigned to different pharmacological classes.

### Non-homogeneous groups of compounds have known common effects

Interesting results can be observed for non-homogeneous groups of compounds for which a predominant pharmacological action could not be assigned. In particular, group G13 (Antiseptics-Estrogens) shows a double effect, clearly visible in Fig. 4, by acting on inflammation-related pathways and on mitosis gene sets. Possible key players of this group are *Safrole*, which has been shown to induce liver DNA damage (explaining the action on stress response pathways) [27] and *Methyl salicylate*, shown to have an estrogenic potential [28], thus increasing the coordinated activity of genes related to cellular replication pathways. Another interesting example is group G7, containing four chemicals with apparently different functions. As suggested by the specific loss of connectivity induced on module “coral1”, related to inflammatory response, the majority of compounds included in this group have antioxidant properties [29–31]. Among these, *Atorvastatin* is a compound belonging to the class of Statins, which was not grouped with the other Statins in G3. Interestingly, no other statins except for one were demonstrated to have antioxidant effects in vitro, confirming the grouping of compounds found with aRI [21, 31].

## Methods

### Data sources

The DrugMatrix [8], available through the Gene Expression Omnibus (GEO) with the accession number GSE57822, contains gene expression profiles from male rat primary tissues (liver, kidney, heart and thigh muscle) and cultured rat hepatocytes, corresponding to treatments with 376 chemicals, and including 994 control samples from rats kept in matched conditions. Each compound was administered at multiple doses and durations (6 h - 7 days), and each combination of tissue, compound, time and dose was profiled in triplicate. Of the 376 chemicals tested, 255 were annotated with either carcinogenicity or genotoxicity information in the Carcinogenic Potency Database (CPDB) [11], corresponding to 3448 profiles. In our study, only the samples from liver were considered, both for controls (279 samples) and for chemical perturbations represented by at least 10 samples and including all doses and durations available. The average sample size used for network inference was of 18 samples, ranging from a minimum of 11 to a maximum of 38 samples, with the corresponding power to detect absolute correlation values of 0.5 with significance below 0.05 ranging from 0.49 to 0.95, respectively.

The Toxicogenomics Project-Genomics Assisted Toxicity Evaluation system (TG-GATEs) [9], is available through ArrayExpress (E-MTAB-800) and includes 21,385 samples of male rat primary liver and kidney tissues, and cultured hepatocytes. TG-GATEs tested 131 chemical compounds. In our validation, only the samples from liver control were used, for a total of 1572 samples used to infer a gene network.

### Data processing

Both Affymetrix datasets were normalized using the R Bioconductor package frma and frmaTools [32]. The Median Absolute Deviation (MAD) was used as the variation filter to select the 7000 best-ranked probes whose expression was then considered for inference of transcriptional networks. Data normalization, gene selection, network inference and other analyses were performed with custom scripts developed using the programming languages R, and several Bioconductor packages.

### Network inference and modules analysis

Transcriptional network inference starts by defining an adjacency matrix *A* = {*a*_*ij*_}, with weight *a*_*ij*_ denoting the strength of the relation of genes *i* and *j* in the expression data. Scale-free transformations (thresholding) can then be applied to the correlation measurements to achieve a scale-free topology typical of biological networks, characterized by relatively few highly connected nodes (hubs) among a larger number of sparsely connected neighbors [1]. In this work, we explored both the direct use of non-transformed correlation networks (CN) as well as of scale-free transformed networks (SFN).

In order to obtain a correlation matrix, Pearson correlation measures between all pairs of gene expression profiles were computed. In the CN approach, the correlation matrix was directly used as the adjacency matrix A. Conversely, in the SFN approach two additional steps were required: i) only those edges with correlation values exceeding a specific threshold were retained, with the threshold selected so that the resulting distribution of connectivities fitted a scale-free topology; and ii) the adjacency matrix *A* was computed by transforming the thresholded correlation values into a topological overlap matrix (TOM), which takes into account the indirect interactions between each couple of genes in the network [1]. In both approaches, hierarchical clustering with Ward’s method was then applied to the obtained adjacency matrix, and a dynamic tree cutting algorithm was applied to determine the number and composition of gene clusters, henceforth referred to as gene modules. We used the R package cutreeDynamic with minimum cluster size set to 10 genes, method set to “hybrid” and “deepSplit” parameter set to 4, which allows a higher number of more homogeneous clusters if compared to other parameter settings. Finally, a Module Differential Connectivity score (MDC) was used to compare the connectivity of gene modules between networks [6]. For a specific module composed of *N* genes and an edge set *EN*, the MDC measures the ratio of the weighted cardinalities of *EN* in the two network, i.e.:

1 $$ M D C\left( X, Y\right)=\frac{\left| E{N}_X\right|}{\left| E{N}_Y\right|} $$

where |*EN*_*X*_| and |*EN*_*Y*_| denote the average connectivities *a*_*ij*_^*X*^ and *a*_*ij*_^*Y*^ among the module’s genes within networks *X* and *Y*. MDC values below 1 represent a loss of connectivity of the module in *X* with respect to *Y*, while values exceeding 1 indicate a gain of connectivity.

### Validation of inference methods

The reproducibility of the network inference procedure was evaluated by two approaches, under the assumption that networks derived from the same experimental conditions should yield very similar structures, with minimal differences in connectivity only due to sampling variability. In the first validation, networks derived from the control (i.e., unexposed) liver samples of two independent datasets, the DrugMatrix (*n* = 279) and the TG-GATEs (*n* = 1572), were constructed and compared. The significance of the overlap between modules extracted from the two networks was assessed by means of the Fisher exact test.

In the second validation, a resampling approach was adopted, whereby the control liver samples from the larger TG-GATEs dataset was randomly split into two equally sized datasets, and corresponding networks were derived and compared, with the procedure repeated 50 times. In both validations, the distributions of MDCs obtained by the two inference approaches (CN and SFN) were computed and compared.

### Inference of Compound and Aggregate Compound Networks

The inference approach (CN) showing the best validation results was used to analyze how different compounds (or groups of compounds) affect the connectivity patterns of specific gene modules. All non-treated liver samples available were used to construct the Control Network, while for the treatment-related networks we relied only on chemical compounds for which at least ten replicate experiments (animals) were available, obtaining 62 Compound Networks.

In order to build Aggregate Networks representing multiple chemical compounds, the similarity of Compound Networks based on their module composition was measured by the adjusted Rand index (aRI) [8]. The aRI is a well-accepted measure that allows for the comparison of clustering results even when these yield different numbers of clusters. Groups of chemical compounds were then identified by applying dynamic tree cutting (cutreeDynamic hybrid, minimum cluster size set to 3 compounds, “deepSplit” set to 4) to the hierarchical tree obtained by merging compounds, with the Ward method, based on their aRI similarity. For each of the 13 groups detected, an aggregate network was built using partial correlation, in place of simple correlation, as the adjacency measure, so as to control for the potential confounding effect of the chemicals grouped. The sample sizes of these groups ranged from 41 to 154, with an average of 86 samples used to infer correlation values. Internal similarity of compounds in each group was assessed by evaluating the overlap of their interacting proteins, as retrieved through the CTD database [33], by means of the Fisher exact test. Specifically, a p-value was obtained for each pair of compounds in a group and the median p-value was used as the score of internal similarity among the aggregated set of compounds. The significance of these measures was assessed by randomly selecting equally sized groups of compounds from the CTD database and computing their internal similarity, with the procedure repeated 1000 times. The same permutation-based approach was used to compute the significance of side effects similarity, this time considering SIDER [15] as a knowledge source to retrieve compound-related information.

### Comparison with other clustering approaches

The grouping obtained with the network-based approach was compared to two alternative methodologies, based on: i) standard clustering methods which do not take advantage of the network structure; ii) information from available network-based knowledge sources.

The first approach starts by defining an average expression profile *M*_*i*_ *=* (*v*_*i*_)*,* for each compound *C*_*i*_, with values *v*_*i*_ obtained as mean expression values for each gene *j* across all samples related to *C*_*i*_. Pairwise distances between compounds *C*_*i*_ and *C*_*k*_ were thus estimated by computing the Euclidean distance of the correspondent profiles *M*_*i*_ and *M*_*k*_.

In the second approach, chemical-protein associations were retrieved through STITCH, a widely adopted public repository of chemical-related networks [14]. STITCH contains multiple evidences of associations, with experimental evidences being one of the most reliable types of recorded interactions. A network with all chemicals and their associated proteins was thus obtained by considering only associations coming from experimental evidences. Subsequently, a distance measure for compounds *C*_*i*_ and *C*_*k*_ was estimated as 1-*J*, *J* being the Jaccard Index of their set of associated proteins *P*_*i*_ and *P*_*k*_,: *J* = *P*_*i*_∩*P*_*k*_ */P*_*i*_∪*P*_*k*_ *.*

In both approaches, the pairwise distances computed were used as input to Hierarchical Clustering (Ward’s method) to obtain compounds groupings.

### Selection and annotation of significant modules

For each Control Network-specific module, a confidence interval for the value of the corresponding MDC with respect to each Aggregate Network (or Individual Compounds network) was computed by randomly selecting with replacement the same number of samples from the replicates of non-treated samples. After 1000 iterations, the standard value of each log-transformed MDC was compared with the obtained estimates of MDC confidence interval. The resulting p-values assess the significance of the deviation of the MDC from 1 (with 1 denoting lack of differential connectivity).

The same bootstrap procedure was adopted for modules extracted from each Aggregate Network (or Individual Compounds network), this time by randomly selecting with replacement the same number of samples from the entire set of perturbations.

In order to rank the control modules most specifically altered by each compound group, the changes in connectivity of each module *m* measured for a compound group *g*_*i*_ with respect to the Control Network *c* were compared to those obtained for the other compound groups [16]. First, the absolute value of the difference in the MDC scores between two groups of compounds g_i_ and g_j_ was computed for each module *m* as:

2 $$ \varDelta {m}_{g i, gj}=\left| \log MD{C}_m\left({g}_i,\  c\right) - \log MD{C}_m\left({g}_j,\  c\right)\right| $$

This was used to compute the specificity of module *m* to compound group *g*_*i*_, as:

3 $$ S p{(m)}_{gi}={\displaystyle {\sum}_{k=1}^N\varDelta {m}_{gi, gk}} $$

where N denotes the total number of compound groups different from *g*_*i*_. For each compound group, modules with score exceeding the top 5th percentile of the overall distribution of specificity scores were selected for enrichment analysis.

Specificity of modules inferred from the Aggregate Compounds Networks was assessed based on two alternative criteria, depending on the frequency of observation of the same module (or a highly overlapping module, Fisher test *p*-value < 0.01) in the networks. Modules identified in more than 50% of the aggregate networks were labeled as “high frequency” and a score *Sp*(*m*)_*gi*_ for module *m* to compound group *g*_*i*_, was computed as described for the Control Network modules (Eqs. 2 and 3). The Specificity score was obtained as $\frac{Sp{(m)}_{gi}}{Ntot}$ where *Ntot* denotes the total number of compound groups. For each compound group, modules with score exceeding the top 5th percentile of the overall distribution of specificity scores were selected as “high frequency” specific modules.

Modules identified in less than 50% of the networks were labelled as “low frequency” and selected based on significance of their correspondent MDC values (*p* < 0.01).

Both Control Network-derived and Aggregate Network-derived (low and high frequency) specific modules were annotated by enrichment of the hallmark gene sets part of the MSigDB compendium [34]. Significance of pathway enrichment was computed using a hyper-geometric distribution-based test and corrected for multiple hypothesis testing across multiple pathway gene sets via the false discovery rate (FDR) estimation. Hallmark pathways with FDR ≤ 0.25 was reported for each specific gene module in both Control Networks and Aggregate Networks.

Selected genes and Hallmark gene sets were compared with the “Perturbational Transcriptome”, a list of genes identified as significantly differentially expressed (with respect to matched controls) in at least five compounds [11]. Each module was tested for enrichment of genes included in the Perturbational Transcriptome by a hyper-geometric test.

### Computational requirements

The pipeline was run on a Linux node with two eight-core 2.6 GHz Intel Xeon processors, and 128 GB RAM. Several R scripts implementing the main steps illustrated in Fig. 1 were run sequentially on the node. Network inference and modules identification (Fig. 1a-b) required approximately 17 s for the Control Network and 9 s for an Individual Compound Network inferred from 20 samples. Pairwise similarity of 62 networks and grouping with the aRI (Fig. 1.c-d) took approximately 15 s. The run time for modules identification and differential connectivity analysis (Fig. 1.e-f) between a 60-module Control Network and a 68-module Aggregate Compound Network was about 6 s, with 0.02 s needed for MDC computation of a module of 100 genes. Similar run times were required for each of the Aggregate Compound Networks. The most computationally intensive part of the pipeline was the significance testing based on bootstrapping, which required repeating correlation and MDC computation 1000 times for each module (Fig. 1.g). Considering an Aggregate Network inferred from 68 samples, this step took on average 1.7 s/iteration, with a total of 28 min required for the complete assessment of MDC q-values, composed of 1000 repetitions. Similarly, significance analysis of Compounds-related modules took 1.8 s/iteration, with a total run time of 30 min needed for q-values estimation. In summary, the Control Network-centered analysis took on average 26.24 min and the Aggregate Network-centered analysis for a single group of chemicals took an average of 28.03 min. It should be noted that once the Control and Aggregate Networks are inferred, the statistical significance tests for the Control Network-centered and each of the Aggregate Network-centered analyses are independent of each other and can thus be executed in parallel.

## Conclusions

We have presented a pipeline for transcriptional network inference and comparison that was primarily designed for the analysis of chemical perturbations from high-throughput transcriptional screening experiments. Here, we applied it to the analysis of gene expression profiles from rat-based chemical exposure experiments. We show that groups of chemicals with similar functions and carcinogenicity/genotoxicity profiles can be identified through our proposed pipeline. In addition, modules with altered connectivity due to the action of specific compounds were enriched for pathways actually related to the chemicals’ action. These findings highlight potential advantages in the application of this network-based approach. In the context of drug discovery (or repositioning), the methods presented here could help assign new functions to novel (or existing) drugs, based on the similarity of their associated network with those built for other known compounds. Additionally, networks with patients as nodes could be compared with the same tools in order to identify groups with a similar response to a set of drugs. In fact, the proposed methodology has broad applicability beyond the uses here described and could be used as an alternative or as a complement to standard approaches of differential gene expression analysis.

As currently implemented, the pipeline identifies modules with differential connectivity irrespective of link directionality. An extension of the method possibly worth investigating would be the modeling of directional links, e.g., obtained by distinguishing between positively and negatively correlated gene pairs. This would require a modification of the connectivity score here adopted, and could potentially highlight modules for which a specific perturbation is able to change the direction of nodes connectivity with respect to a reference condition. On the other hand, the obtained results would likely become more difficult to interpret and annotate.

## Additional files

Additional file 1: **Supplementary Figures.** Additional figures with supplementary results. (PDF 1229 kb)

Additional file 2: Table S1. Global differential connectivity (GDC) of the entire networks inferred for compound groups with respect to the Control Network. (XLSX 8 kb)

Additional file 3: Table S2. Control Network-related modules with connectivity specifically altered by compound groups. (XLSX 12 kb)

Additional file 4: Table S3. Aggregate Network-related modules with connectivity specifically altered by compound groups. (XLSX 12 kb)

Additional file 5: Table S4. Comparison of genes and gene sets identified as differentially connected in the network-based approach and by the standard differential expression analysis. (XLSX 8 kb)

## Abbreviations

aRI: Adjusted Rand Index
CN: Correlation network
MDC: Module Differential Connectivity
SFN: Scale free network
