# Supplementary material for: Network-based analysis of transcriptional profiles from chemical perturbations experiments
Source: BMC Bioinformatics. 2017 Mar 23;18(Suppl 5):130. doi: 10.1186/s12859-017-1536-9 (PMC5374700; doi:10.1186/s12859-017-1536-9)
Supplement: Supplementary file 1 — Supplementary Figures. Additional figures with supplementary results. (PDF 1229 kb) [file 12859_2017_1536_MOESM1_ESM.pdf]

Supplementary Figures

Network-Based Analysis of Transcriptional Profiles from Chemical Perturbations Experiments

F. Mulas, A. Li, D. Sherr, S. Monti

Figure S1

Figure S2

Figure S3

Figure S4

Figure S5

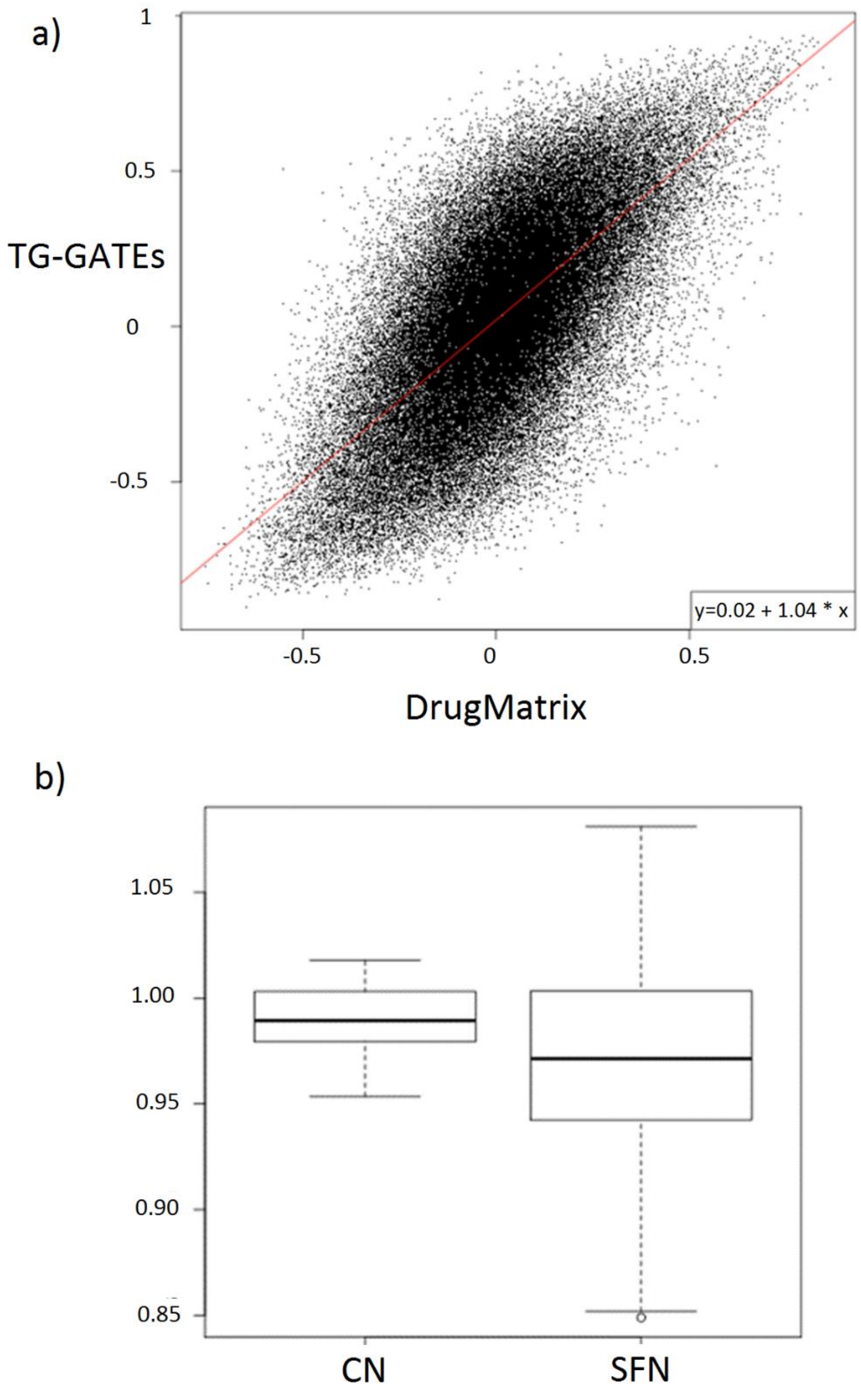

Figures S1. **Validation of network inference and comparison.** a. Ranges of gene correlation values in DrugMatrix and TG-GATEs datasets. b. Distribution of MDC values obtained comparing modules from the same data set (TG-GATEs) and conditions (liver control samples), using Scale-free approaches (SFN) or non-thresholded Correlation Networks (CN).

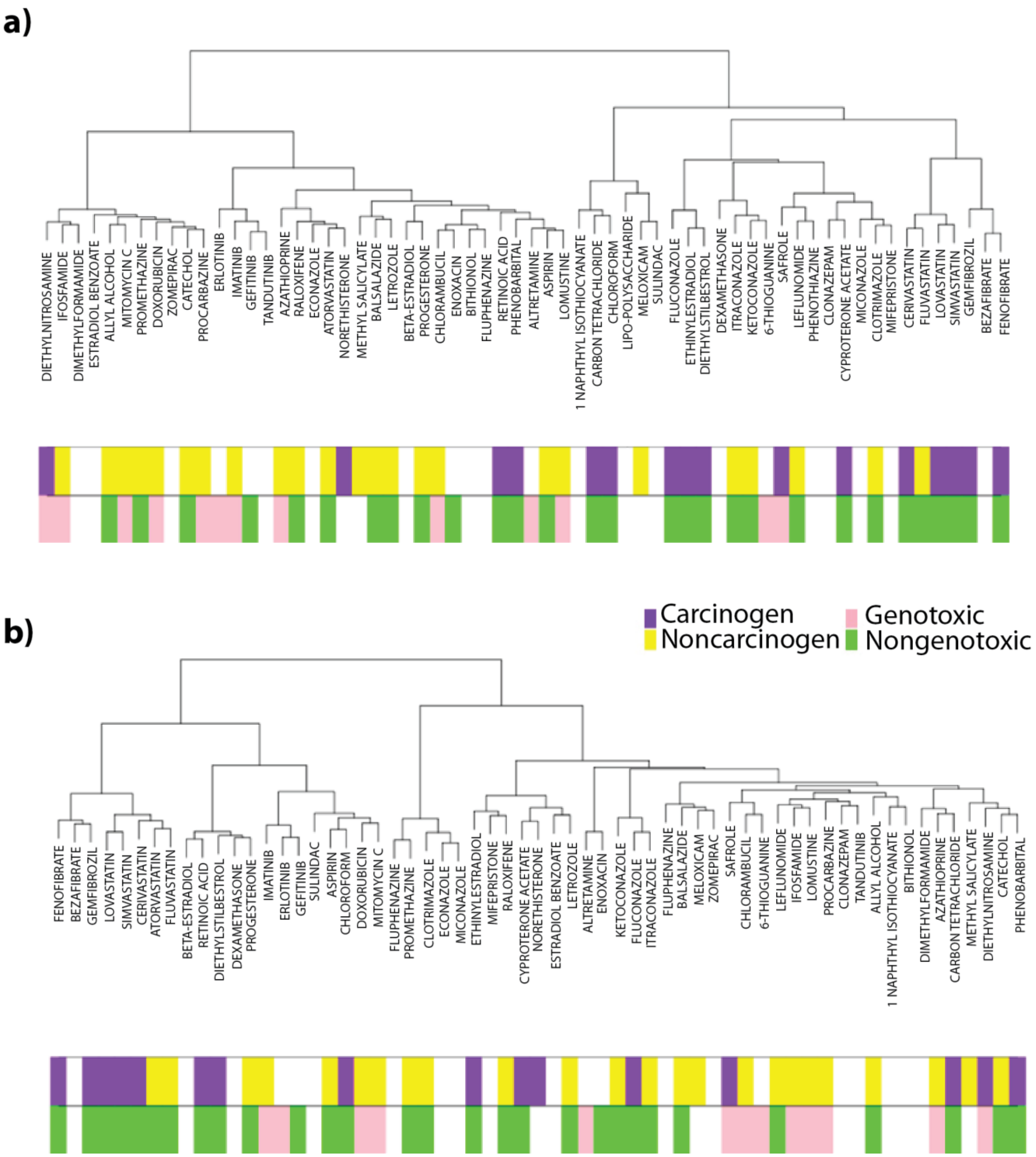

Figure S2. Comparison with other grouping approaches. Grouping of 62 compounds based on their average gene expression profile (a) or on their interacting proteins retrieved through public databases (b).

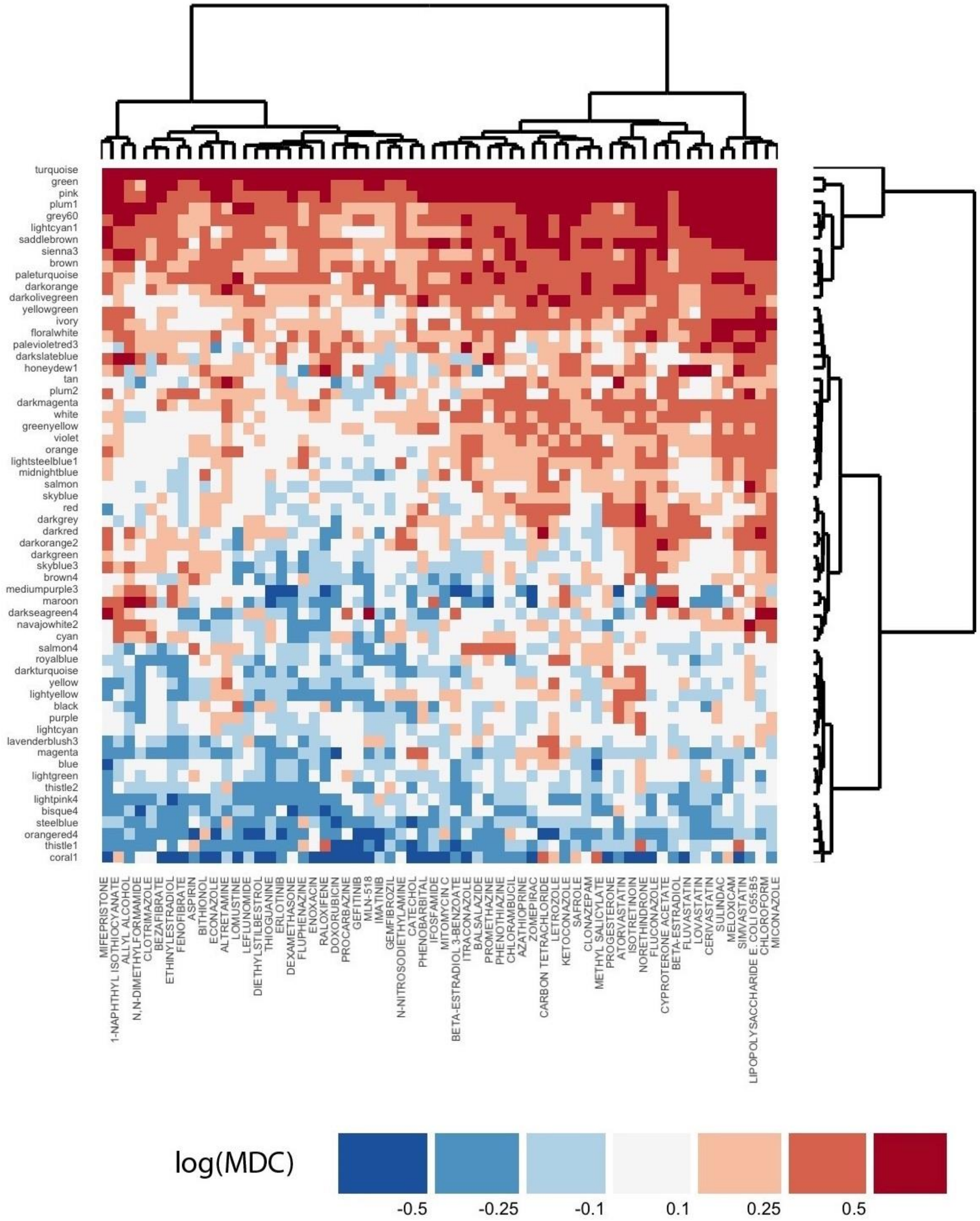

Figure S3. **Differential connectivity of Control modules in *Individual chemicals*-related networks.**

Differential connectivity of 60 Control modules (rows) induced by 62 chemical compounds (columns). **a.** MDC values, with blue and red indicating a loss and a gain of connectivity, respectively.

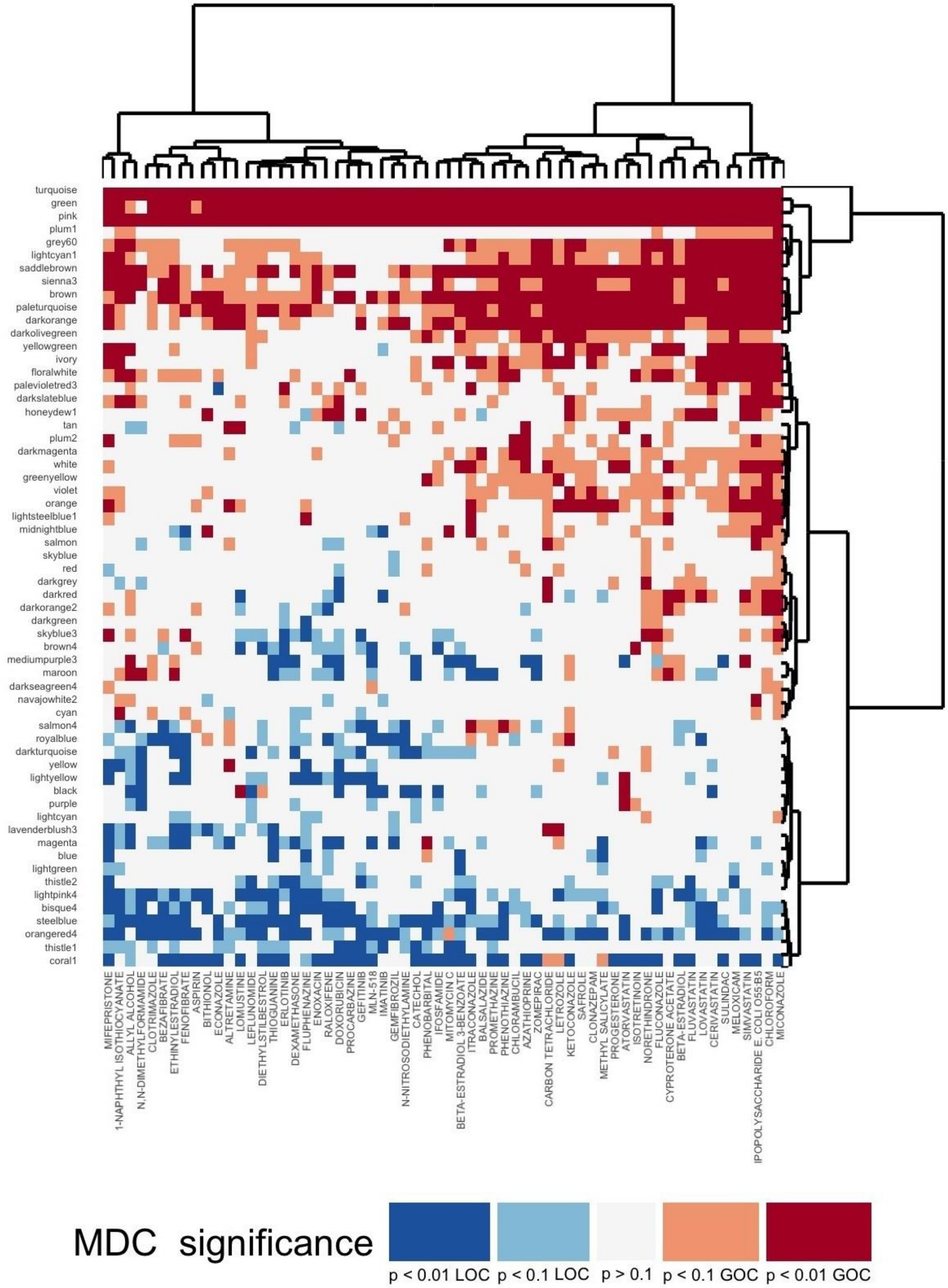

Figure S3.b. Significance level (q-values) of the module differential connectivity. Dark blue and dark red indicate more significant loss and gain of connectivity, respectively.

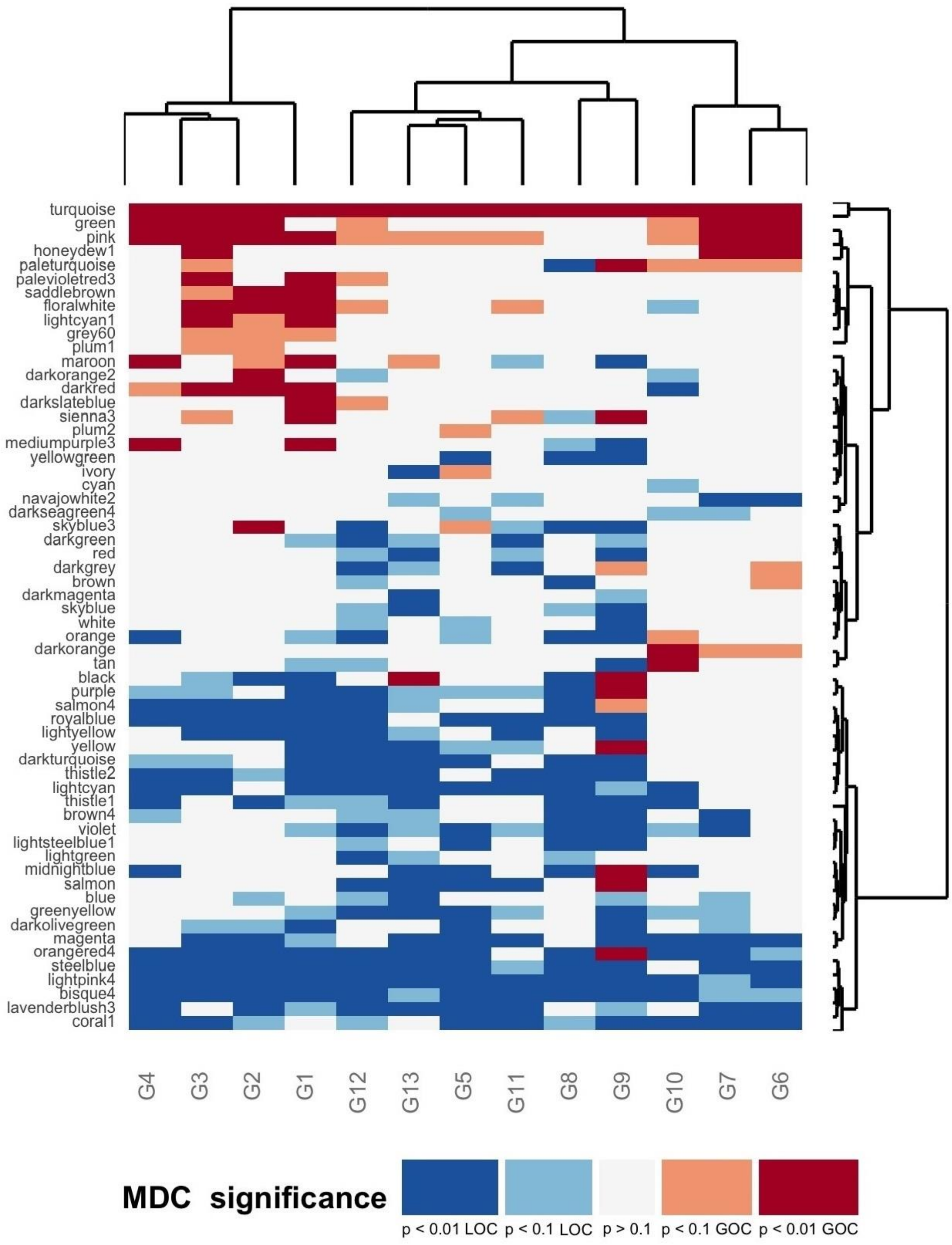

Figure S4. **Significance of MDC values in *Aggregate Compounds networks*.**
Significance of the differential connectivity values of 60 Control modules (rows) induced by 13 groups of compounds (columns). The heatmap is color-coded according to the significance level (q-values) of the corresponding MDC values. Dark blue and dark red indicate more significant loss and gain of connectivity, respectively.

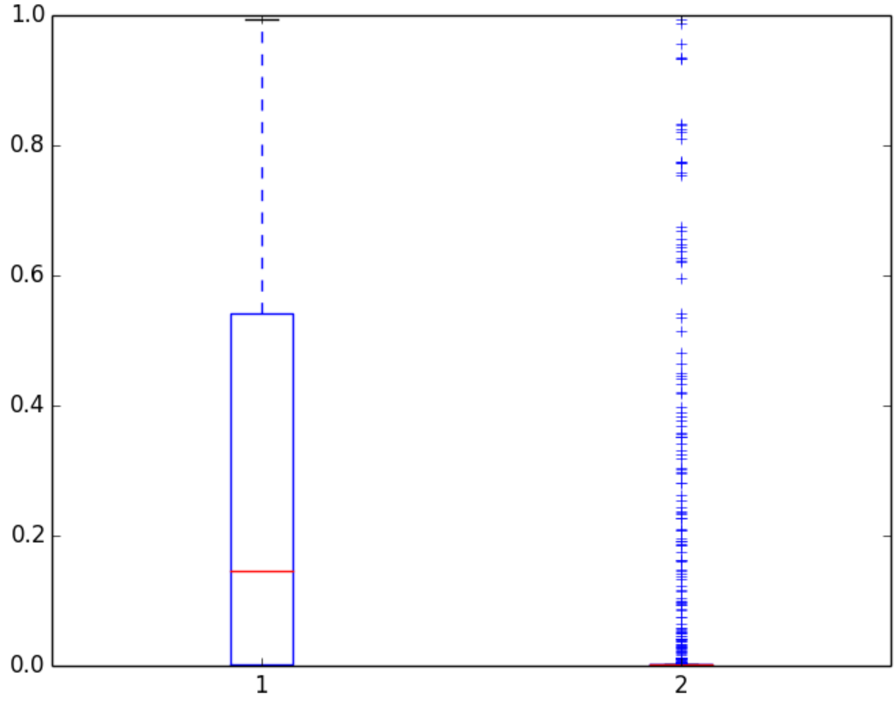

Figure S5. P-value distribution obtained with the bootstrap approach (1) and with a gene permutation strategy (2).
